# HEART-HEALTHY LIFESTYLES REDUCE THE RISK OF COGNITIVE DECLINE

**DOI:** 10.1093/geroni/igac059.212

**Published:** 2022-12-20

**Authors:** Sarah Lock, Kristine Yaffe

**Affiliations:** AARP, Washington, District of Columbia, United States; University of California, San Francisco, San Francisco, California, United States

## Abstract

The Global Council on Brain Health (GCBH) convened experts from around the world to examine the impact of cardiovascular risk factors on brain health. GCBH issue experts carefully considered how high blood pressure, diabetes and stroke, as well as lifestyle choices such as diet and exercise, influence brain health in adults age 50 and over. The GCBH concluded that the best evidence to date shows us “what’s good for the heart is good for the brain.” Their consensus puts powerful tools in the hands of adults wanting to protect their brain as they age. The GCBH adopted 10 recommendations for individuals to incorporate into their lives to keep heart and blood vessels healthy and reduce the risk for cognitive decline and dementia. These recommendations and 16 practical tips are provided in the final report. Liaisons from numerous civic and nonprofit organizations with expertise in heart and brain health helped develop these recommendations for adults 50+ and their health care providers.

